# Comparing the Artificial Intelligence Detection Models to Standard Diagnostic Methods and Alternative Models in Identifying Alzheimer’s Disease in At-Risk or Early Symptomatic Individuals: A Scoping Review

**DOI:** 10.7759/cureus.75389

**Published:** 2024-12-09

**Authors:** Britty Babu, Gauri Parvathy, Fathima S Mohideen Bawa, Gurnoor S Gill, Jeeya Patel, Dataar S Sibia, Jayadev Sureddi, Vidhi Patel

**Affiliations:** 1 Medicine, Tbilisi State Medical University, Tbilisi, GEO; 2 Medicine, Tbilisi state medical university, Tbilisi, GEO; 3 Medicine, Florida Atlantic University Charles E. Schmidt College of Medicine, Boca Raton, USA; 4 Medicine/Science, American Heritage High School, Delray Beach, USA; 5 Medicine/Science, Florida Atlantic University High School, Boca Raton, USA; 6 Medicine, Comprehensive Blood and Cancer Center, Bakersfield, USA; 7 Information Technology, Gandhinagar University, Moti Bhoyan, IND

**Keywords:** alzheimer’s disease, artificial inteligence, diagnostic method, geriatric models of care, neurology and critical care

## Abstract

Alzheimer's disease (AD) and other neurodegenerative illnesses place a heavy strain on the world's healthcare systems, particularly among the aging population. With a focus on research from January 2022 to September 2023, this scoping review, which adheres to Preferred Reporting Items for Systematic Reviews and Meta-Analysis extension for Scoping Reviews (PRISMA-Scr) criteria, examines the changing landscape of artificial intelligence (AI) applications for early AD detection and diagnosis. Forty-four carefully chosen articles were selected from a pool of 2,966 articles for the qualitative synthesis. The research reveals impressive advancements in AI-driven approaches, including neuroimaging, genomics, cognitive tests, and blood-based biomarkers. Notably, AI models focusing on deep learning (DL) algorithms demonstrate outstanding accuracy in early AD identification, often even before the onset of clinical symptoms. Multimodal approaches, which combine information from various sources, including neuroimaging and clinical assessments, provide comprehensive insights into the complex nature of AD. The study also emphasizes the critical role that blood-based and genetic biomarkers play in strengthening AD diagnosis and risk assessment. When combined with clinical or imaging data, genetic variations and polygenic risk scores help to improve prediction models. In a similar vein, blood-based biomarkers provide non-invasive instruments for detecting metabolic changes linked to AD. Cognitive and functional evaluations, which include neuropsychological examinations and assessments of daily living activities, serve as essential benchmarks for monitoring the course of AD and directing treatment interventions. When these evaluations are included in machine learning models, the diagnosis accuracy is improved, and treatment monitoring is made more accessible. In addition, including methods that support model interpretability and explainability helps in the thorough understanding and valuable implementation of AI-driven insights in clinical contexts. This review further identifies several gaps in the research landscape, including the need for diverse, high-quality datasets to address data heterogeneity and improve model generalizability. Practical implementation challenges, such as integrating AI systems into clinical workflows and clinician adoption, are highlighted as critical barriers to real-world application. Moreover, ethical considerations, particularly surrounding data privacy and informed consent, must be prioritized as AI adoption in healthcare accelerates. Performance metrics (e.g., sensitivity, specificity, and area under the curve (AUC)) for AI-based approaches are discussed, with a need for clearer reporting and comparative analyses. Addressing these limitations, alongside methodological clarity and critical evaluation of biases, would strengthen the credibility of AI applications in AD detection. By expanding its scope, this study highlights areas for improvement and future opportunities in early detection, aiming to bridge the gap between innovative AI technologies and practical clinical utility.

## Introduction and background

Neurodegenerative diseases (NDs) are a leading cause of disability in the older population [1]. Alzheimer's disease (AD) is the most common neurodegenerative disorder and ranks as the sixth leading cause of death in the United States. The number of new cases of AD and other dementias is expected to triple by the year 2050, reaching a staggering 152 million cases. This projection implies that there will be approximately one new case of dementia every three seconds globally, highlighting the magnitude of the issue [2]. AD is a chronic, progressive neurodegenerative disorder and the most prevalent cause of dementia, contributing to 60-80% of all cases globally. Pathologically, AD is defined by the abnormal accumulation of extracellular amyloid-beta (Aβ) plaques and intracellular neurofibrillary tangles composed of hyperphosphorylated tau protein. These aggregates trigger widespread synaptic dysfunction, neuronal loss, and brain atrophy, particularly affecting the hippocampus and cortical regions critical for memory and executive function [1,2].

Clinically, AD typically begins with mild cognitive impairment, such as difficulty with short-term memory and complex problem-solving, and progresses to severe dementia involving significant cognitive deficits, language impairments, and loss of motor coordination. In advanced stages, patients lose the ability to perform basic activities of daily living, requiring full-time care. Current diagnostic approaches rely on neuroimaging techniques (e.g., MRI, PET scans), cerebrospinal fluid (CSF) biomarkers like Aβ42/40 ratios and tau proteins, and cognitive testing to confirm the disease’s presence [1,2]. Although there is no cure, treatments such as cholinesterase inhibitors (e.g., donepezil, rivastigmine) and N-methyl-D-aspartate (NMDA) receptor antagonists (e.g., memantine) aim to temporarily alleviate symptoms and slow progression. Emerging research into biomarkers and targeted therapies holds promise for earlier diagnosis and more effective interventions [1,2].

AD's global disease burden is projected to reach $2 trillion by 2030. This underlines the immense economic impact of the disease on a worldwide scale, making it a significant public health concern. Despite extensive research and advances in clinical practice, diagnosing AD accurately remains a challenge. Less than 50% of AD patients are diagnosed accurately for their pathology and disease progression based solely on their clinical symptoms. This indicates the need for improved diagnostic methods and early detection approaches. In fact, dementia, including AD, is projected to reach 152 million by 2050, with the greatest increases in low- and middle-income countries [3].

The presence of amyloid plaques and neurofibrillary tangles in histopathology is considered the most conclusive evidence for AD. Amnestic mild cognitive impairment (aMCI) is an early stage of dementia characterized by minor issues with memory, speech, or decision-making. aMCI is widely recognized as a precursor to AD. It represents an intermediate stage of cognitive decline wherein individuals demonstrate significant deficits in memory and related cognitive domains, but these impairments do not yet interfere substantially with their ability to carry out daily activities. Critically, aMCI serves as a key diagnostic and therapeutic window; more than 80% of individuals with aMCI progress to AD within six years [4]. This makes identifying and intervening during this stage pivotal for delaying or potentially altering disease progression. What makes aMCI significant is that more than 80% of individuals who meet the criteria for aMCI tend to progress to AD within six years [4].

The presence of amyloid plaques and neurofibrillary tangles in histopathology is considered the most conclusive evidence for AD. However, it is noteworthy that these features may be more prominent in the later stages of the disease. The early onset of AD is not necessarily correlated with plaques but instead with synaptic and neuronal loss. This highlights the importance of detecting subtle changes in brain structure and function to identify early stages of the disease [3]. However, the diagnosis of AD has not been standardized, and the primary diagnostic methods include magnetic resonance imaging (MRI) and positron emission tomography (PET) brain imaging, biochemical analysis of Aβ42/40, and total tau (t-tau) and phosphorylated tau (p-tau181) levels in the CSF [5].

Early and accurate diagnosis of AD is crucial for several reasons. First, early detection allows for preventive measures and interventions that may slow disease progression or delay its onset. Moreover, timely diagnosis enables the early initiation of appropriate treatments and care, improving the disease's management and enhancing the patient's quality of life. Second, accurate diagnosis aids in tracking the development of the disease, allowing healthcare professionals to provide tailored care and support to patients and their caregivers [2].

The increasing development of neuroimaging techniques, such as MRI, diffusion tensor imaging (DTI), and PET, has revitalized the study of human brain structure and function. Researchers have been using these imaging modalities individually or in combination to classify AD and explore its different stages [6]. With advancements in technology and the increasing volume of brain-imaging data, machine learning (ML) and deep learning (DL) have become essential for accurately analyzing and predicting AD based on brain-imaging data [2].

Artificial intelligence (AI) and ML are transformative technologies that enable computers to analyze complex datasets and uncover patterns that would be challenging or impossible for human interpretation alone. In AD research, these tools are particularly valuable for analyzing large-scale neuroimaging data, genetic profiles, and cognitive assessments. By identifying subtle biomarkers and integrating data from multiple sources, AI and ML provide opportunities for earlier, more precise diagnosis of AD compared to traditional methods. For example, DL algorithms can detect abnormalities in MRI or PET scans that signal early AD pathology, often before clinical symptoms become apparent [5,6]. ML's main advantage is its ability to learn from intricate datasets and identify hidden non-linear relationships. ML algorithms can provide data-driven classifications in multidimensional spaces, unlike traditional hypothesis-driven approaches that test subsets of predictors. 

Traditional diagnostic methods for AD, such as neuroimaging and cognitive testing, depend heavily on clinician expertise and often yield inconsistent results, particularly in detecting early-stage AD. In addition, the sheer complexity and volume of diagnostic data, including multimodal imaging and biomarker profiles, exceed the analytical capacity of traditional approaches. AI and ML address this gap by offering tools capable of synthesizing diverse datasets to enhance diagnostic accuracy, efficiency, and reproducibility. Advancements in biotechnology have led to the reliable recording of various aspects of human biology, such as genetic data and biomarkers like cerebral blood flow and brain imaging. This has resulted in vast biological datasets from which ML algorithms can learn to classify participants or predict predefined classes. Integrating genetic data with other modalities can introduce complexity beyond the human capacity to process and analyze impartially. As a result, ML plays a crucial role in handling and extracting valuable insights from these diverse and intricate datasets [6].

Due to these complexities, improving the accuracy of early diagnosis for AD and MCI remains a significant challenge. Researchers continue to explore advanced ML, DL techniques, and multimodal approaches that combine different imaging modalities to address these challenges and achieve more accurate and early detection of cognitive impairments [6].

Methods

This scoping review follows the reporting guidelines as outlined in the Preferred Reporting Items for Systematic Reviews and Meta-Analysis extension for Scoping Reviews (PRISMA-Scr) [7].

Search

Relevant articles published (from 2021 to 2023) were searched in databases (PubMed). Studies before the year 2021 were not searched as the expertise of AI has evolved considerably. The following Medical Subject Headings (MeSH) terms were searched in databases: (("Alzheimer's" OR "dementia") AND ("machine learning" OR "deep learning"))). 

We chose to exclude studies published before 2021 to ensure the review focused on the most recent advancements in AI and ML applications for AD diagnosis [5]. The field of AI is rapidly evolving, with significant breakthroughs in DL architectures, multimodal data integration, and explainable AI occurring in recent years. By focusing on studies published from January 2021 onward, we aimed to capture the state-of-the-art methodologies and their clinical relevance, reflecting the current landscape of AI-driven diagnostic tools [6].

Older studies might rely on outdated techniques or less sophisticated AI models, which do not leverage the computational and algorithmic advancements achieved in the past few years. In addition, many older studies may not account for the increased availability of high-quality datasets or the latest ethical and regulatory considerations in AI implementation [6]. By narrowing our scope to more recent years, we ensured the review provided timely insights and addressed the current challenges and opportunities in applying AI to AD diagnosis [6].

This review scoped the available literature to answer the following questions: What is the accuracy of AI-based diagnostic methods for the early detection of AD in patients at risk of the condition? What is the efficiency of AI-based diagnostic methods for the early detection of AD in patients at risk of the condition? Regarding early detection of AD in patients at risk, how does the efficiency of AI-based diagnostic methods compare to traditional diagnostic methods?

Eligibility Criteria

Eligibility criteria were enlisted before the study commenced. A scoping review does not report all published literature; rather, it presents a snapshot picture of the current status, highlights gaps in knowledge, and suggests ways to improve further research.

The articles described AI applications to aid the detection of dementia/AD. Prospective/retrospective studies and articles written in English and published from January 2022 to September 2023 were included.

All review articles, books, editorials, and studies not in English that do not have clearly specified classification results and research conducted on animals were excluded. Filters were used to identify papers written in English between 2022 and 2023. Three independent reviewers reviewed the studies. Study characteristics were identified based on population (patients at risk of AD), intervention (AI-based diagnostic methods), comparison (traditional diagnostic methods), and outcome (accuracy and efficiency for early detection).

Study Selection

**Figure 1:**
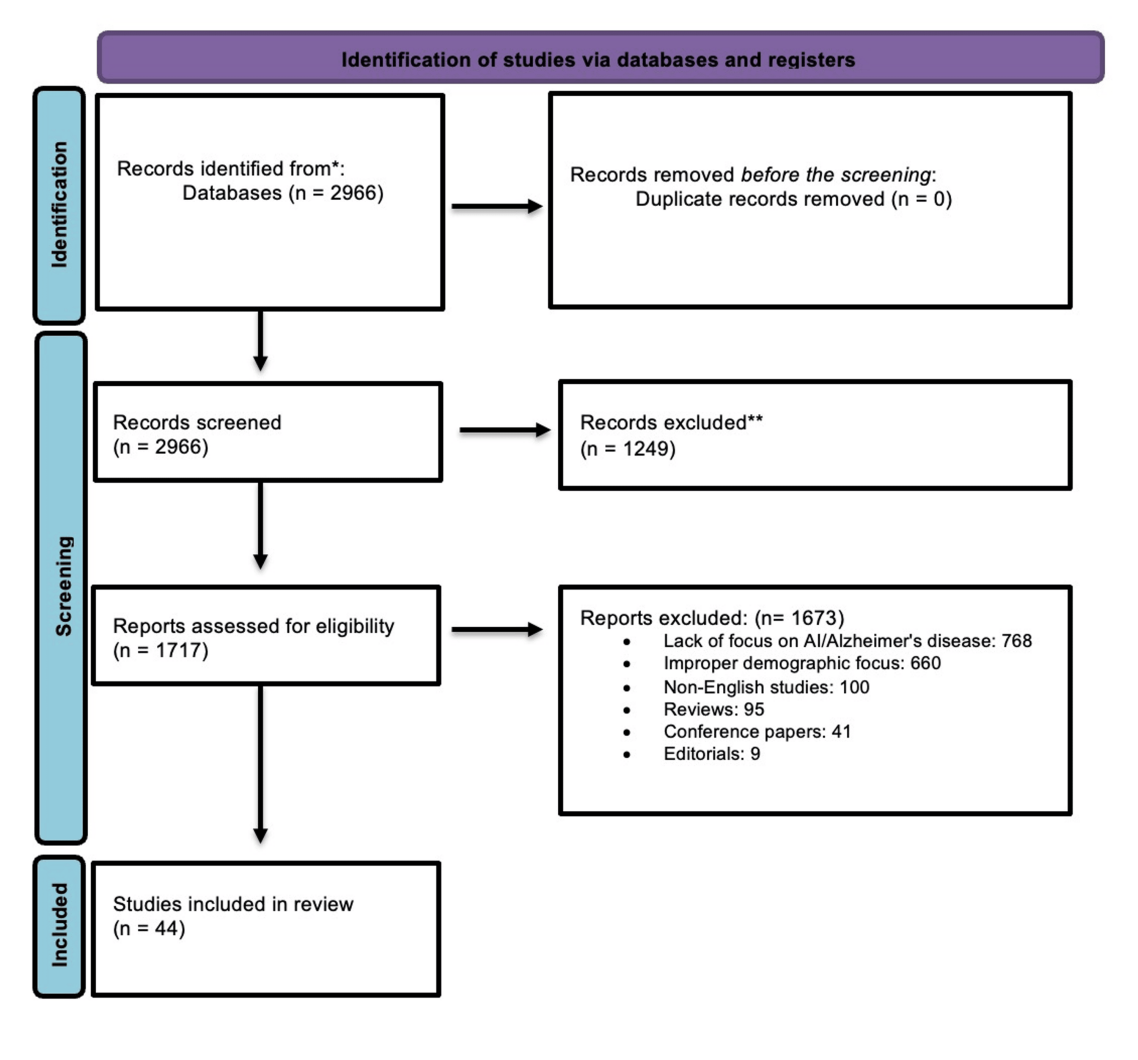
Flow chart for the study selection

A total of 2,966 articles were identified after using the MeSH mentioned above terms in PubMed search after excluding books, editorials, and reviews. No duplicates were found. After meticulously excluding articles lacking full text and studies not explicitly focused on AI and AD, 1,717 full-text articles were shortlisted and assessed for eligibility. Forty-four articles were finally included for qualitative synthesis, as mentioned in Figure 1.

Sources of Evidence

A thorough evaluation procedure was carried out in an extended effort to comprehend the efficacy of diagnostic techniques for AD in those at risk or exhibiting early signs. In the beginning, 2,966 studies were located in the PubMed database. A number of exclusion criteria were used to ensure that only the most relevant and instructive research was included. The absence of complete articles in 1,249 research, the classification of 95 as reviews, 12 as conference papers, three as editorials, and six as animal studies were among the grounds for elimination. In addition, 660 research were disqualified for not being in the English language, and 768 studies were disqualified for having an improper demographic focus. Forty-four articles were chosen as a result of the thorough screening process and satisfied the strict requirements for admission.

This scoping review includes a wide variety of research findings on AI models for AD prediction. To detect AD across multiple cohorts, this research makes use of a variety of detection methods and prediction models. With some research comprising hundreds of participants and others using smaller datasets, sample sizes might vary greatly. The methods of detection include EEG signals, cognitive tests, retinal images, genetic information, and neuro-imaging modalities including MRI and PET scans [4,8,9]. DL algorithms, ML classifiers, and ensemble approaches are among the prediction models used, highlighting the fact that research on AD is interdisciplinary [10,11]. The studies include a wide range of methodologies, such as methodological studies, retrospective case-control studies, computational research, and diagnostic validation.

Others emphasize genetic markers or functional brain imaging for early diagnosis. By contrast, other research emphasizes using multimodal DL for dementia evaluation or utilizes retinal images as a diagnostic tool [12,13]. Sample sizes range from a few hundred to thousands, allowing for a thorough assessment of the performance of AI models across various datasets. These research predictive models demonstrate the adaptability of AI techniques for predicting AD by including a broad range of ML algorithms, such as decision trees, support vector machines, convolutional neural networks, and ensemble methods [2,14]. The papers also include various study types, from computational research to diagnostic validation, emphasizing the numerous strategies researchers use to improve early diagnosis and risk prediction of AD.

## Review

Relevant outcomes

A summary of the 44 studies included throughout this scoping review can be found in Table 1.

**Table 1:** Comprehensive summary of machine learning and artificial intelligence applications in Alzheimer's disease diagnosis and progression prediction CNN: convolutional neural network, MRI: magnetic resonance imaging, MCI: mild cognitive impairment, AD: Alzheimer's disease, APOE: apolipoprotein E, ML: machine learning, EEG: electroencephalogram, cfDNA: cell-free deoxyribonucleic acid, PET: positron emission tomography, FDG: fludeoxyglucose-18, OASIS: Open Access Series of Imaging Studies, KNN: K-nearest neighbor, AUC: area under the curve, DAT: dementia of AD type, VEGF-A: vascular endothelial growth factor A, LSR: lesion-to-spinal cord signal intensity ratio, AUROC: area under the ROC curve, rs-fMRI: resting state task-based fMRI, Smile-GAN: semi-supervised clustering GAN, SCD: subjective cognitive decline, SVM: support vector machine, PCA: principal component analysis, LASSO: least absolute shrinkage and selection operator, NLP: natural language processing, SbRNS: sandpiper-based recurrent neural system Table credits: Gurnoor Gill

Author(s)	Key findings	Unique characteristics
GA Aguayo et al., 2023 [1]	Machine learning predicts neurodegenerative diseases in older populations.	TabTransformer model applied in a cohort study.
D AlSaeed et al., 2022 [2]	CNN-based MRI feature extraction achieved high AD classification accuracy.	Automated CNN applied to neuroimaging datasets.
Rye A et al., 2022 [3]	Predictive modeling for MCI to AD conversion using biomarkers.	Biomarkers validated via APOE and hippocampal volume.
N J Herzog et al., 2021 [4]	Brain asymmetry features identified for AD diagnosis.	Neuroanatomical asymmetry is used in ML classification.
H Yu, 2021 [5]	Platelet biomarkers linked to cognitive decline using proteomics.	Proteomic markers revealed novel pathways for AD.
J Sheng et al., 2022 [6]	Genetic and imaging data achieved 98% classification accuracy.	Multimodal diagnostic approach for AD.
T W Rowe et al., 2021 [8]	Life-time risk prediction models for AD were reviewed systematically.	Focused on ML models’ predictive performance.
N Chedid et al., 2022 [9]	EEG-based ML pipeline achieved 81% accuracy for AD.	Artifact-free EEG data processing validated.
CY Cheung et al., 2022 [10]	Retinal biomarkers linked with AD detection achieved 83.6% accuracy.	Retinal imaging is proposed as a non-invasive diagnostic tool.
RO Bahado-Singh et al., 2022 [11]	cfDNA methylation patterns identified for early AD detection.	Highlighted epigenetic mechanisms as biomarkers.
PR Millar et al.,2023 [12]	Brain age biomarkers linked to cognitive decline and AD stages.	Multimodal MRI imaging combined with functional biomarkers.
M Odusami et al., 2022 [13]	ML framework achieved high accuracy for early AD detection.	DenseNet and ResNet integrated with MRI.
L Chiricosta et al., 2022 [14]	Blood transcriptome data linked oxidative stress to AD pathology.	Transcriptomics-based machine learning predictive models developed.
A A et al., 2022 [15]	Automated PET neuroimaging enhanced AD early-stage detection.	CNN frameworks applied to PET imaging.
I Beheshti et al., 2022 [16]	FDG-PET imaging tracked MCI progression to AD.	PET scans were validated for metabolic activity metrics.
X Wang et al., 2022 [17]	Retinal biomarkers correlated with cognitive decline.	Eye-based imaging is proposed as a scalable diagnostic method.
A Taylor 15 al., 2022 [18]	Longitudinal imaging patterns predicted AD-related neurodegeneration.	Combined brain age biomarkers with explainable AI models.
C Kavitha et al., 2022 [19]	Gradient boosting models improved AD early-stage prediction.	ML classifiers applied to OASIS datasets.
JB Toledo et al., 2022 [20]	SPARE-Tau indices predicted cognitive decline stages in AD.	Imaging biomarkers validated for clinical utility.
Y M Elgammal et al., 2022 [21]	Multifractal KNN achieved superior classification accuracy in AD MRI imaging.	Geometry-based classification methods validated for AD.
Diogo et al., 2022 [22]	Multi-diagnostic ML approach for AD using MRI, achieving 90.6% accuracy in distinguishing healthy controls and AD.	Hippocampal features as major contributors; generalizable across datasets and MRI protocols.
Gao et al., 2023 [23]	PRS and EHR data predicted AD with an AUC of 0.88 in a large UK Biobank cohort.	PRSs for age-at-onset were more predictive than age; novel feature importance patterns were identified.
Mirabnahrazam et al., 2022 [24]	Combined MRI and genetic data for DAT prediction showed improved performance in distinguishing DAT progression.	Novel multimodal stratification method for DAT; detailed analysis of imaging and genetic contributions.
Sekaran et al., 2023 [25]	Identified the ORAI2 gene as a blood-based biomarker for AD using explainable AI.	AI-identified STIM1 and TRPC3 as interacting genes linked to AD progression.
Petrelis et al., 2022 [26]	VEGF-A-related genetic variants were found to protect against AD progression.	A model with epistatic interactions between VEGF-A, APOE, and LSR demonstrated 72% accuracy.
Feng et al., 2023 [27]	Blood-based metabolic pathway signatures developed for non-invasive AD diagnosis.	Distinct AD subgroups identified with varying metabolic and immune profiles; achieved AUC of 0.99 in validation.
Vik et al., 2023 [28]	Subtle changes in daily functioning predicted AD progression from MCI with 70% accuracy.	Informant-reported functional activity levels and verbal memory were highlighted as key predictors.
Kobayashi et al., 2022 [29]	Multi-task drawing tests improved early AD detection to 75.2% accuracy.	Combined drawing data captured multiple cognitive impairments; automated analysis scalable to non-specialist settings.
Zhang et al., 2021 [30]	3D CNN and ensemble learning achieved 95.2% accuracy in AD vs. normal controls.	Innovative data denoising module; high performance in MRI-based AD diagnosis.
Feng et al., 2022 [31]	Deep learning outperformed other biomarkers for prodromal AD detection (AUROC = 0.788).	Model localized hippocampal activation; reduced patient burden and cost through non-invasive MRI.
Alorf and Khan, 2022 [32]	Multi-label classification of six AD stages from rs-fMRI using graph convolutional networks achieved 84.03% accuracy.	Identified key brain regions (e.g., frontal gyrus) for differentiating AD stages.
Yang et al., 2021 [33]	Smile-GAN identified subtypes of neurodegeneration and predicted AD progression pathways.	Semi-supervised clustering via GAN offered precision diagnostics and informed clinical trials.
Guan et al., 2023 [34]	Attention-guided autoencoder accurately predicted SCD progression to MCI or AD.	Leveraged domain transfer learning for small datasets and localized disease-specific brain regions.
Lai et al., 2022 [35]	Identified immune subtypes and genes linked to AD pathology using explainable machine learning.	CXCR4, PPP3R1, HSP90AB1, CXCL10, and S100A12 genes are linked to distinct immune subtypes of AD.
Prasad VK et al., 2024 [36]	CNN achieved 99.29% accuracy for Alzheimer’s diagnosis using MRI data.	Proposed a cloud-based framework integrating preprocessing, execution, and diagnostic layers, emphasizing scalability and data security.
Mofrad et al., 2021 [37]	Combined cognitive trajectories and MRI features to predict AD conversion from MCI with improved accuracy.	Longitudinal mixed-effects modeling for both cognitive and structural brain measures.
Tian et al., 2021 [38]	Retinal vasculature features provided an alternative biomarker for AD diagnosis with 82.44% accuracy.	Highlighted the utility of retinal imaging as non-invasive and cost-effective.
Bron et al., 2021 [39]	Validated cross-cohort generalizability of SVM and CNN for AD classification, showing comparable performance.	Demonstrated robustness in external validation with similar AUC scores for both models.
Muñoz-Castro et al., 2022 [40]	Machine learning models identified distinct astrocyte and microglia states in AD and normal aging.	Multiplex fluorescent immunohistochemistry captured spatial relationships with AD pathology.
Kim et al., 2022 [41]	Deep learning (VUNO Med-DeepBrain) achieved 87.1% accuracy in diagnosing AD with 2D brain MRI.	Improved sensitivity (93.3%) and specificity (85.5%) compared to medical experts; scalable for non-specialist settings.
Li et al., 2022 [42]	Proposed a classification framework for complex, imbalanced data using functional PCA and group LASSO for early AD.	Achieved high sensitivity for longitudinal and high-dimensional data; focused on early detection challenges in AD.
Liu et al., 2022 [43]	Developed a transfer learning model using speech and NLP for early AD detection, achieving 88% accuracy.	Combined pre-trained DistilBERT and logistic regression for robust language feature extraction and binary classification.
Swarnalatha, 2023 [44]	Proposed SbRNS model for EEG-based AD severity prediction with precision and recall above conventional methods.	Addressed noise in EEG signals with advanced pre-processing; classified severity into low, medium, and high categories.
Bogdanovic et al., 2022 [45]	XGBoost model analyzed 12,000+ subjects, yielding 0.84 F1 score and explainable insights into AD diagnosis.	Used Shapley values for feature importance; focused on gender, APOE4, and age as key diagnostic predictors.

Understanding AD and enhancing early diagnosis are both made possible by imaging technologies. By offering high-resolution structural images of the brain using MRI, ML models like 3D convolutional neural networks (CNNs) can detect AD-related biomarkers and patterns with remarkable accuracy [15]. Positron emission tomography (PET), which uses FDG-PET imaging, has also proved crucial in determining alterations in brain metabolism linked to AD [16]. Researchers have progressed in AD categorization using DL methods and dimension-reduced 2D image analysis. In addition, optical coherence tomography (OCT), which measures the peripapillary retinal nerve fiber layer (pRNFL) and macular thickness, as well as retinal imaging, notably via retinal photos, have emerged as possible methods for identifying AD-related changes [17]. ML algorithms, feature selection, and cross-validation have unlocked these imaging techniques' potential to assist in early AD diagnosis.

In the effort to find the early signs of AD, these imaging techniques serve as crucial cornerstones. In addition to improving accuracy, using ML algorithms to analyze data from MRI, FDG-PET, retinal photos, and OCT provides effective and non-invasive diagnostic tools [4,17]. These discoveries offer invaluable insights into AD pathogenesis and open the door to more potent treatments and therapies for this crippling neurodegenerative disease, which has enormous potential for both physicians and researchers.

In our drive to improve diagnosis accuracy and fully comprehend this complicated neurodegenerative disorder, multi-modal techniques in AD prediction constitute a significant advancement [17]. These methods are prudent because they recognize the complexity of AD, which includes various pathological and clinical aspects. These methods aim to provide a more comprehensive understanding of the condition by combining information from many sources, such as neuroimaging, clinical evaluations, and genetics [19]. Combining neuroimaging data from MRI and PET scans with clinical data from demographic and cognitive test results is one such approach [20]. With the help of this fusion, structural brain alterations and cognitive function may be captured, leading to more accurate and thorough forecasts of AD [21].

Another crucial component of multi-modal techniques is imaging fusion, which uses information from imaging modalities like MRI and PET scans to create a coherent picture of the changes in the brain caused by AD [22]. With the help of this fusion, the diagnostic accuracy of various imaging modalities will be increased by using their complementing properties. Researchers may further explore the genetic mechanisms underlying the pathophysiology of AD by combining genetic data, such as gene expression profiles or polygenic risk scores, with neuroimaging data [23]. This method reveals a more thorough grasp of the disease's underlying mechanics. In the end, multi-modal techniques supported by ML ensemble methods clinical and imaging biomarkers result in enhanced diagnostic performance, encouraging possibilities for early identification and customized therapy approaches suited to the wide range of AD patients [24].

The study of genetic and blood-based biomarkers has become an essential area of study in AD, providing vital information on the pathophysiology and diagnosis of this neurodegenerative disease [23,25]. The four alleles are linked to higher vulnerability to the illness and genetic biomarkers, notably changes in genes like APOE, have been identified as critical indications of AD risk [3]. Multiple genetic variations were combined to create polygenic risk scores, which have shown promise in predicting the risk and development of AD even before clinical symptoms appear. The creation of more precise prediction models for AD diagnosis has been made possible by merging genetic data with clinical or imaging data. These genetic indicators are promising for early intervention and individualized therapy approaches that enhance clinical results [26].

On the other hand, blood-based biomarkers have become popular as non-invasive instruments for identifying alterations linked to AD [25]. Studies of blood plasma samples have shown metabolic abnormalities related to AD development, such as modifications in the pathways for oxidative phosphorylation and fatty acid production [27]. To accurately predict the risk of AD, ML models incorporating polygenic risk scores, electronic medical records, and blood-based biomarkers have been created [23]. In addition, when combined with ML, specific platelet proteins and measures from retinal scans have shown promise in assisting AD diagnosis [5]. These developments improve diagnostic precision and provide opportunities for tracking disease progression, assessing the effectiveness of treatments, and identifying those more susceptible to developing AD. As a result, genetic and blood-based biomarkers have become crucial components of AD research with positive clinical implications.

The examination of AD includes cognitive and functional tests, which are crucial for determining how this neurodegenerative disorder affects a person's mental and physical capacities [27]. Neuropsychological tests, which evaluate memory, executive function, language, and visuospatial abilities, have repeatedly shown that AD patients exhibit severe cognitive deficits [29]. One defining characteristic is memory impairment, especially in episodic memory. The Mini-Mental State Examination (MMSE) and the Alzheimer's Disease Assessment Scale-Cognitive Subscale (ADAS-Cog) are commonly used tests that measure cognitive loss and enable the separation of various phases of AD, such as moderate cognitive impairment (MCI) and mild AD [17]. These tests are essential for tracking illness development and early diagnosis.

On the other hand, functional evaluations concentrate on a person's capacity to carry out everyday duties and activities independently. Functions, including dressing, bathing, cooking, and money management, are assessed using tools like the activities of daily living (ADL) and instrumental activities of daily living (IADL) scales. Research shows that functional skills gradually deteriorate as AD worsens [30]. These evaluations are necessary to comprehend how the condition affects a patient's quality of life, to direct treatment plans, and to evaluate the efficacy of therapies. In addition, they support doctors in assessing the degree of physical restrictions and cognitive impairments, allowing for the prompt implementation of individualized care plans and therapies. In research settings, functional and cognitive data are incorporated into machine learning models to improve the accuracy of AD diagnosis and prediction, making them essential tools for assessing treatment effectiveness and monitoring disease development over time [1].

In AD research, evaluating prediction performance is crucial, especially when using ML models and algorithms for early diagnosis and intervention. These models include DL algorithms, ensemble models, support vector machines, and decision trees, among other ML methods [19]. To predict AD, they use a variety of input variables, including neuroimaging data (MRI and PET scans), genetic data, cognitive test results, and clinical assessments.

The capacity of these models to identify AD at an early stage, sometimes before clinical symptoms appear, is one of their outstanding accomplishments. Researchers evaluate the models' ability to discriminate between AD patients and healthy controls or those with MCI using performance measures including accuracy, sensitivity, specificity, and the area under the receiver operating characteristic curve (AUROC) [16,31]. Studies that differentiate between healthy people, those with MCI, and various stages of AD use multi-class categorization to provide a more thorough evaluation of cognitive loss [32].

Adding new information, notably neuroimaging data, has significantly improved prediction performance. Integrating many modalities, including genetic information, cognitive test results, and different imaging modalities, has resulted in improved accuracy and resilience in AD prediction. For these models to be clinically valuable, high prediction performance is essential since it allows for early intervention and individualized treatment programs. Strong models may also be used to identify people more likely to develop AD, allowing for the development of precision medicine and the facilitation of preventative actions [13].

There are still issues, such as the need for thorough validation of different datasets to guarantee the generalizability and dependability of the models. Problems like data heterogeneity and sample size restrictions must be addressed for more accurate forecasts [33].

When using ML models in AD research, explainability and interpretability are crucial. Models are often divided into two groups: transparent models, which explain the reasoning behind forecasts, and black-box models, which make accurate predictions but lack transparency [9]. Techniques like feature significance analysis and technologies like SHapley Additive exPlanations (SHAP) and Local Interpretable Model-agnostic Explanations (LIME) have been used to improve interpretability. These methods help discover biomarkers and close the gap between clinical practice and research by revealing the critical elements affecting AD classification and development. Clinicians may test model robustness across various patient groups, diagnose early AD indications, and customize therapies and treatments based on patient-centric findings. Ultimately, explainability and interpretability enable doctors and researchers to make well-informed choices, accelerate the hunt for biomarkers, and improve the treatment and outcomes for AD patients [13,34].

Explainability and interpretability are crucial characteristics of machine learning models in AD research. These models may be transparent, allowing insights into the reasoning behind forecasts, or black-box, delivering precise predictions with no transparency. To improve interpretability, researchers use methods such as feature importance analysis, SHAP, LIME, and others [35]. This aids in discovering pertinent biomarkers, promotes therapeutic relevance, and allows for the customization of therapies for particular patients. Transparent models assist in risk assessment, promote generalization across various patient groups, and aid in the early diagnosis of AD [21]. In the end, explainability and interpretability increase confidence in machine learning models and shed light on the underlying processes of AD, aiding in developing new biomarkers and bettering patient treatment, as shown in Table 2.

**Table 2:** Comparative performance of AI and conventional diagnostic methods in early Alzheimer's detection across various data modalities AI: artificial intelligence, MRI: magnetic resonance imaging, ML: machine learning, RF: random forest, LR: logistic regression, SVM: support vector machine, Smile-GAN: semi-supervised clustering, GAN: generative adversarial network, GBM: glioblastoma multiforme, SVM: support vector machine, CNN: convolutional neural network, HC: healthy control, AD: Alzheimer's disease, NC: normal control, EMCI: early mild cognitive impairment, EEG: electroencephalography, KNN: K-nearest neighbor, LDA: latent Dirichlet allocation, NLP: natural language processing, SMOTE: synthetic minority over-sampling method, FDG-PET: fluorodeoxyglucose-positron emission tomography, MCI: mild cognitive impairment, rs-fMRI: resting-state functional MRI, SSAE: stacked sparse autoencoder, BC-GCN: brain connectivity graph convolutional network, LS-SVM-RBF: least-squares support-vector machines-radial basis function, SVM: support vector machine, LSTM: long short-term memory, DSNet: detect-to-summarize network, FGAN: federated generative adversarial network, CSF: cerebrospinal fluid, EHR: electronic health record, SHAP: Shapley additive explanation Table credits: Britty Babu

Technique	AI model/algorithm used	Key findings/results
MRI and cognitive tests	Fusion model, MRI-only model, non-imaging model	The fusion model outperformed MRI-only and was comparable to the non-imaging model.
Retinal photographs	Not mentioned	Promising results in Alzheimer's detection using retinal vasculature analysis and ML
Gene expression (brain tissues)	LightGBM, CatBoost, XGBoost, RF, LR, SVM	Identified immune genes associated with Alzheimer's using ML tools
MRI data (Smile-GAN)	Smile-GAN	Identified neurodegeneration patterns for precision diagnostics and trial recruitment
Brain section (astrocytes and microglia)	Spectral clustering, GBM	ML models classified astrocytes and microglia with high accuracy.
Fresh blood	scikit-learn Python package	Four platelet proteins yielded promising results for predicting cognitive decline.
Cognitive and MRI images	Ensemble model (various ML algorithms)	Improved classification of healthy controls vs. cognitive impairment with MRI features
MRI data	Decision tree, random forest, SVM, gradient boosting, voting classifiers	Proposed a classification scheme that achieved high accuracy in Alzheimer's diagnosis
MRI images	3D CNN, gradient-boosting model	The deep learning model identified predictive imaging biomarkers for early detection.
T1-weighted brain MRI scans	3D CNN, sigmoid output	Outperformed other neuroimaging biomarkers in Alzheimer's diagnosis
T1-weighted brain MRI scans	VUNO Med-DeepBrain AD (DBAD)	DBAD supported non-specialized physicians in diagnosing AD.
MRI images	Linear SVM, decision tree, random forest, etc.	High-performance classifiers distinguished HC and AD patients.
Various data types (XGBoost)	XGBoost, random forest	XGBoost algorithm outperformed random forest in classifying neurodegenerative diagnoses.
Retinal images	Not mentioned	Promising performance in Alzheimer's detection using feature selection and cross-validation
Brain MRI and genetic data (MRI and genetic data)	Supervised ensemble learning	Combining MRI and genetic data improved DAT prediction.
MRI images	Various machine learning algorithms	Classify different stages of dementia including NC, EMCI, and AD
EEG	Decision trees, SVM, KNN, LDA	A transparent and explainable ML approach enhanced AD diagnosis.
Speech and NLP	DistilBERT with various classifiers	Logistic regression with DistilBERT showed the best performance.
MRI images	3D CNN	High accuracy was achieved in AD diagnosis using a CNN-based model.
Cognitive assessments	Random forest, multi-classifier network, SMOTE	Combining MCI and AD as a single class improved screening test effectiveness.
FDG-PET imaging	Not mentioned	Successfully differentiated AD patients from those with MCI using PET scans
rs-fMRI	SSAE model and BC-GCN	Identified significant brain regions for AD classification
MRI data	LS-SVM-RBF, SVM, KNN, random forest	LS-SVM-RBF achieved higher accuracy, and KNN showed better sensitivity.
Cognitive test data and FDG-PET analysis	Mono-objective and multi-objective evolutionary algorithms	Designed an explainable AI framework for diagnosing neurodegenerative diseases
Structural MRI	SVM, CNN, LSTM	SVM provided good performance on small-sample-sized datasets.
PET and MRI	Deep learning framework (DSNet, FGAN)	Achieved state-of-the-art performance in AD identification and MCI conversion prediction
CSF and plasma	Machine learning with cross-validation	Predicted CSF biomarkers in cognitively normal subjects
Structural and FC-MRI data	Three machine learning algorithms	Functional and structural MRI models capture complementary signals.
PET and MRI	CNN	Achieved high accuracy in classifying AD and NC patients based on 18FDG-PET images
Polygenic risk scores and EHRs	XGBoost and SHAP	Predicted the risk of developing AD using polygenic risk scores and electronic health records
EEG and amyloid PET scans	Various machine learning models	EEG-based biomarkers can predict future cognitive impairment in preclinical AD.
Metabolic hallmarks in blood plasma	XGBoost, Boruta, random forest, etc.	Identified metabolic pathways correlated with AD and divided patients into subgroups
Structural MRI	Support vector machine, random forest, KNN, LDA	Demonstrated good performance, interpretability, and speed on small-sample-sized datasets
Combination of brain imaging and genetic data analysis	Linear SVM	Achieved good classification accuracy by selecting relevant features
PET and MRI	Deep learning framework (DSNet, FGAN)	Achieved state-of-the-art performance in AD identification and MCI conversion prediction
EEG signal analysis	Recurrent neural system (SbRNS)	Classified AD severity ranges using EEG signals
Biomarker (multichannel fluorescent sensor array)	Simplified sensor array and ML algorithm	Demonstrated the power of multichannel signals in clinical detection via ML
Biomarker	Multimodal deep learning model (MDLCN)	MDLCN outperformed baseline models for gene interaction prediction in specific cell types.
MRI images	Multifractal geometry, ML classification techniques	Achieved high accuracy and specificity in AD classification using MRI images
SPARE-Tau index	Machine learning-derived SPARE-Tau	SPARE-Tau showed strong associations with cognitive scores and disease progression.
Drawing tests and cognitive tests	SVM, K-nearest neighbors, random forest	Features from multiple drawing tasks improved the automated detection of AD and MCI.
MRI images	Deep learning models (ResNet, DenseNet, EfficientNet, MAE, DeiT)	Visual transformer model DeiT outperformed CNN models for AD classification.
DNA analysis (peripheral blood)	Elastic net (EN) model	Predicted CSF biomarkers in cognitively normal subjects
Cell-free DNA (peripheral blood)	Not mentioned	Utilized machine learning for clinical detection using a sensor array with multichannel signals
Real data application	Machine learning models (logistic regression, decision trees, SVM, etc.)	A promising approach for disease screening

Discussion

Millions of people worldwide are afflicted with the crippling neurological ailment known as AD. For prompt intervention and treatment, a rapid and correct diagnosis is essential. By contrasting their precision, sensitivity, specificity, and predictive value with accepted diagnostic procedures and competing prediction models, this scoping review seeks to understand the function of AI prediction models in detecting AD. The scoping review examined several research papers using AI methods and data sources, including neuroimaging (MRI, PET), cognitive tests, genetic information, blood-based biomarkers, retinal pictures, and EEG signals.

The scoping study included a wide range of data, highlighting how AI may be used to forecast AD. The researchers also used support vector machines, DL architectures like CNNs, and boosting approaches [36]. These models showed promise for transforming early AD diagnosis and progression tracking. The crucial significance of neuroimaging, which includes MRI and PET scans, became apparent when AI models outperformed conventional diagnostic techniques in recognizing AD based on complex brain patterns [36]. The multimodal integration of data from several sources, including genetics, cognitive tests, retinal imaging, and blood-based biomarkers, improved prediction accuracy and revealed fresh insights into AD [27,38].

In addition, non-invasive methods using blood- and retinal-based biomarkers showed great promise and provided reasonably priced alternatives to invasive imaging methods. ML models can identify people with AD, MCI, and healthy people, making cognitive evaluations an essential diagnostic tool [17,39]. Its promise as a non-invasive method of monitoring cognitive decline was highlighted by the contribution of EEG signal processing to predicting AD severity. Hub genes associated with the course of AD were revealed through genetic data combined with ML, revealing prospective treatment targets [25]. Building confidence in AI-driven diagnostics requires transparency and interpretability, which explainable AI (XAI) methodologies emphasized as being essential. By revealing subtle insights, temporal trajectory analysis and multimodal imaging improved AD identification and progression prediction [22]. These results imply that AI-powered prediction models have enormous potential to revolutionize the early diagnosis and treatment of AD.

Clinical evaluations and cognitive tests, which are the mainstays of conventional diagnostic techniques, may only sometimes provide the degree of precision needed, particularly for an early diagnosis [36]. Contrarily, AI models using transformer-based topologies and convolutional neural networks (CNNs) have shown astounding accuracy in identifying AD. These AI models can process and evaluate complicated data, such as brain MRI pictures, EEG signals, or genetic information, to find minor patterns linked to the condition. An essential benefit of AI models is their improved accuracy [40].

Furthermore, AI models have excellent specificity and sensitivity for detecting AD. They are exceptional at spotting small indicators and trends that may result in rapid and precise diagnosis. On the other hand, standard diagnostic techniques may sometimes attain a different degree of sensitivity and specificity since they sometimes rely on arbitrary clinical observations and cognitive tests [40]. Variability in these established techniques may lead to delayed or incorrect diagnosis.

In addition, AI models have remarkable predictive potential, especially when identifying those at risk of acquiring AD in the prodromal or preclinical phases. Early diagnosis allows for prompt therapies, prospective lifestyle changes, and clinical trial participation, all of which may be essential for controlling the course of the condition. Instead of forecasting the progression of the illness, traditional diagnostic techniques are often more suited for diagnosing AD patients who have already been diagnosed [22].

The capacity of AI models to combine and interpret multimodal data from many sources, offering a more thorough understanding of AD, is a remarkable benefit. They may integrate data from MRI scans, genetic markers, cognitive tests, and other sources to improve diagnostic precision and better understand the complexity of the illness. Traditional approaches, on the other hand, often depend on isolated modalities or clinical tests, which cannot fully encapsulate AD [42].

Many AI models also include excellent interpretability characteristics that help physicians comprehend the logic behind a given diagnosis [43]. This openness promotes trust and makes incorporating AI into therapeutic practice easier. By contrast, conventional techniques' decision-making processes might need to be clarified, making it difficult to justify a diagnosis, particularly in complicated circumstances [31].

Finally, the availability of large and varied datasets for training and validation is essential to developing AI models. These datasets are also essential for creating and generalizing models. Traditional approaches, on the other hand, depend more on clinical judgment and evaluations than large datasets, which may restrict their ability to glean insights from data [38].

To improve AD diagnosis, researchers have explored various prediction models, including traditional statistical methods, biomarker-based approaches, and hybrid models combining multiple data sources [24]. AI models, particularly DL networks, excel at handling complex, high-dimensional data, such as brain MRI images, genomics, and natural language processing, identifying intricate patterns that traditional methods may overlook due to data simplification or feature reduction [42,43].

A key advantage of AI is its ability to seamlessly integrate diverse data types, such as imaging, genetics, cognitive tests, and clinical evaluations, improving diagnostic accuracy [23]. Unlike traditional models that require labor-intensive feature engineering, AI can automatically select features, enabling better adaptability to large datasets. However, biomarker-based models often rely on predefined markers and may miss emerging AD indicators [44].

AI models outperform traditional methods in predictive accuracy, sensitivity, and specificity, particularly for early-stage AD diagnosis [6,11]. However, their complexity and "black box" nature pose challenges to interpretability, although recent efforts focus on creating more explainable models [13,45]. Traditional statistical models, while less accurate, often offer greater transparency, aiding clinical decision-making.

Finally, AI models depend heavily on large, diverse datasets for training and validation, which raises concerns about data accessibility, privacy, and quality [21]. By contrast, alternative models may require less data, making them suitable for resource-limited settings but potentially less robust.

The use of AI to diagnose AD has enormous therapeutic potential. First and foremost, AI enables early and accurate AD diagnosis, considerably enhancing patient outcomes by allowing prompt interventions and individualized treatment regimens based on unique patient data. In addition, it supports appropriate therapy modifications, improves long-term care methods, and monitors the development of the condition [28]. AI's ability to combine many data sources gives us a more complete picture of AD, improving diagnostic precision and enabling patients to lead brain-healthy lives.

In addition, using AI in AD diagnosis assists caregivers by lessening their load and raising the standard of care. Beyond its use in medicine, AI speeds up research into AD and the creation of new drugs by selecting candidates for clinical trials and allowing telemedicine and remote patient monitoring to improve patient access to treatment [24]. To retain trust and keep ethical standards, however, the appropriate use of AI in AD diagnosis calls for careful consideration of moral issues, notably data protection and informed permission, as AI continues to develop and become a crucial aspect of AD diagnosis and treatment.

Integration of AI Models Into Clinical Workflows and Their Role in Diagnostic Methods

The integration of AI-driven diagnostic tools into existing clinical workflows offers significant opportunities to enhance the diagnosis and management of AD. AI models, particularly those leveraging multimodal data fusion, demonstrate diagnostic accuracy and predictive capabilities that often surpass traditional methods [6,12,20,37]. These models can be integrated into clinical workflows in several impactful ways. First, they can function as clinical decision support systems (CDSSs), serving as adjunct tools to assist clinicians by providing risk assessments and diagnostic probabilities based on multimodal inputs, such as neuroimaging, genetic markers, and cognitive test results [7,12,19,30]. Such systems streamline clinical decision-making by offering detailed and interpretable insights [21,34,43]. In addition, AI algorithms can facilitate early screening and risk stratification by identifying high-risk individuals through non-invasive techniques like retinal imaging or blood-based biomarkers [5,16,24]. By complementing routine screening protocols, these tools enable the prioritization of patients for further diagnostic evaluations [6,9,18]. Moreover, AI can optimize workflows by automating data analysis, such as processing MRI scans or interpreting genetic profiles, which reduces the workload on clinicians and allows for more focused patient care [2,10,12,36]. Furthermore, AI systems can integrate seamlessly with electronic health record (EHR) platforms, flagging abnormal patterns or suggesting follow-up actions, thereby ensuring continuity of care and improving the management of patient populations [22,27,42].

AI models have the potential to either complement or, in specific scenarios, replace traditional diagnostic modalities. In complementary roles, AI-based predictions from modalities such as retinal imaging or EEG signals can act as secondary verification steps for diagnoses derived from neuroimaging or cognitive tests [4,6,12,29]. This multimodal approach enhances diagnostic robustness and minimizes the risk of errors [9,15,25,33]. In resource-limited settings, where access to advanced imaging technologies such as MRI or PET scans is restricted, AI-driven blood biomarker analysis or portable EEG-based diagnostic tools can serve as standalone alternatives [5,16,24]. These models provide cost-effective and scalable solutions, bridging the gap in healthcare accessibility and enabling earlier interventions in underserved areas [7,20,37].

While the benefits of integrating AI into clinical workflows are substantial, several challenges must be addressed to ensure successful implementation. Validation of AI models across diverse populations is critical to guarantee their generalizability and effectiveness [13,23,30]. Ethical considerations, particularly those surrounding data privacy and security, are paramount in building trust in AI systems [21,28,41]. Moreover, ensuring clinician adoption of AI recommendations requires efforts to enhance the interpretability of these models [12,31,34]. Transparent AI systems that provide insights into their decision-making processes are essential to overcoming skepticism and achieving regulatory compliance [18,22,43].

The transformative potential of AI in AD diagnosis is evident in its ability to reshape diagnostic paradigms, offering tools that complement and, in some cases, replace traditional approaches. From early detection to workflow optimization and scalable diagnostic solutions, AI promises to significantly enhance the accuracy, efficiency, and accessibility of AD diagnosis [6,9,19,27]. However, addressing challenges such as model validation, ethical concerns, and interpretability will be crucial to realizing the full potential of AI in clinical practice [13,23,38,44].

Limitations

AI and ML face several limitations in detecting AD, which impact their reliability, generalizability, and integration into clinical practice. One significant challenge is data heterogeneity and limited sample size. AI models often struggle with the variability in data sources, including differences in imaging modalities, demographic information, and biomarkers. This lack of uniformity, coupled with small and non-diverse datasets, reduces the models' ability to generalize across populations, limiting their effectiveness in real-world scenarios. Ensuring access to large, diverse, and high-quality datasets remains a pressing issue.

Another major limitation is the interpretability and explainability of AI models. Many AI systems, especially those based on DL, are considered "black boxes," making it difficult to understand or justify their predictions. This lack of transparency creates a significant barrier to clinical adoption, as clinicians need interpretable outputs to make informed decisions and build trust in the technology. Efforts like feature importance analysis and interpretability tools such as SHAP (SHapley Additive exPlanations) and LIME (Local Interpretable Model-agnostic Explanations) aim to address this issue, but these solutions are still in development and not yet widely implemented. Ethical and regulatory challenges also hinder AI applications in AD diagnosis. The use of patient data for training these models raises concerns about privacy, security, and compliance with stringent regulations like GDPR (General Data Protection Regulation) and HIPAA (Health Insurance Portability and Accountability Act). Balancing the need for large datasets with ethical data-handling practices remains a complex issue, especially as data-sharing practices vary across regions and institutions. Validation and generalizability pose further challenges. AI models require rigorous validation across diverse datasets to ensure consistent performance in different populations and healthcare settings. Without this validation, models may yield biased or unreliable results, particularly in underrepresented groups such as minorities or those from low-resource settings. This issue underscores the need for comprehensive testing to ensure equity in diagnostic accuracy and outcomes. Integration into clinical workflows also presents a barrier. For AI to be widely adopted, it must seamlessly integrate with existing healthcare systems and provide user-friendly interfaces for clinicians. Skepticism among healthcare providers regarding the reliability of AI-generated recommendations further complicates this process. Transparent and easily interpretable outputs are necessary to foster trust and ensure the models complement, rather than disrupt, clinical decision-making.

Moreover, the complexity of processing multimodal data is another limitation. While AI has the potential to combine imaging, genetic, and cognitive assessment data to improve diagnostic accuracy, this integration introduces significant computational and algorithmic challenges. Effective handling of multimodal data requires advanced strategies and substantial computational resources, which are often inaccessible to many researchers and healthcare institutions. Performance variability across diagnostic modalities is another challenge for AI in AD detection. The accuracy of AI models can vary significantly depending on the modality used, such as MRI, PET, or retinal imaging. This inconsistency makes it difficult to standardize results and ensure reliable diagnostic outcomes across different approaches, creating a hurdle for broader implementation. The cost and resource requirements of developing and deploying AI models are substantial. Training AI systems demands significant computational power and technical expertise, which may not be feasible in resource-constrained environments. This limitation is particularly problematic for low- and middle-income countries, where scalable diagnostic solutions are most needed. Addressing these resource constraints is essential for equitable access to AI technologies in healthcare.

When assessing the results of the scoping review on AI-based diagnostic techniques for AD, it is vital to consider some notable limitations. First and foremost, there are issues with the review's capacity to accurately reflect the whole research landscape in this quickly changing subject due to its relatively small sample size of 45 publications from the PubMed database from January 2022 to September 2023. Due to this restriction, significant papers that were published in other databases or outside the predetermined period may have been excluded, perhaps leading to an inadequate overview of AI applications in AD diagnosis. Also, this study relies solely on PubMed for article retrieval, omitting other key databases like Scopus, Web of Science, and IEEE Xplore, thereby limiting this review's comprehensiveness.

PubMed's emphasis on English-language papers also introduces a possible language and database bias since pertinent research undertaken in other languages or housed on alternative platforms may have yet to be noticed. Another restriction is the lack of animal research since the context they may have offered for developing AI-based diagnostic techniques for AD could have been beneficial.

In addition, the scoping review lacks another essential component of scoping reviews: a rigorous evaluation of research quality. It is difficult to evaluate the methodological rigor and dependability of the included publications when such quality evaluations are absent, which might undermine the overall validity of the review's conclusions. It is also difficult to compare and extrapolate the included research findings because of the wide variation in study design, AI models, data sources, and diagnostic methodologies. It highlights the need to exercise care when extrapolating general findings from such a diverse set of studies.

Moreover, although several studies presented encouraging findings, the evaluation should be aware of any possible restrictions on generalizability to larger populations and contexts. Future studies should examine how well AI-based diagnostic techniques can be adapted to various healthcare settings since real-world applicability may vary greatly from controlled research conditions. The review's other noteworthy flaws include the potential for publication bias, the lack of specifics on its approach, and the crucial role data quality plays in the effectiveness of AI models. Future research in this area must prioritize using diverse and high-quality datasets for model development, testing AI systems in real-world clinical settings, dealing with ethical issues relating to data privacy and bias, and conducting longitudinal studies to accurately measure predictive accuracy and patient outcomes.

## Conclusions

This scoping review offers a comprehensive overview of the diverse array of ML and AI models employed in AD detection and prediction. The review encompasses extensive data sources, from neuroimaging to genetic information, cognitive assessments, and innovative techniques such as retinal photography and EEG signals. Notably, fusion models that integrate both imaging and non-imaging features emerge as a key finding, consistently demonstrating superior performance in classifying cognitive status and detecting AD. This underscores the importance of adopting a holistic approach that leverages various data modalities to enhance diagnostic accuracy. Furthermore, the review underscores the transformative impact of ML and AI on AD research and diagnosis, offering the potential for early detection and predictive capabilities for disease progression monitoring. Ethical considerations, including data privacy and transparency, are highlighted as crucial as AI advances in healthcare. Despite these promising insights, the review acknowledges the challenges, such as data quality issues and the need for robust clinical validation. It also calls for further exploration of interpretability, multimodal data fusion, and the development of more transparent AI models.
